# Screening and Improving the Recombinant Nitrilases and Application in Biotransformation of Iminodiacetonitrile to Iminodiacetic Acid

**DOI:** 10.1371/journal.pone.0067197

**Published:** 2013-06-27

**Authors:** Zhi-Qiang Liu, Peter James Baker, Feng Cheng, Ya-Ping Xue, Yu-Guo Zheng, Yin-Chu Shen

**Affiliations:** Institute of Bioengineering, Zhejiang University of Technology, Hangzhou, People’s Republic of China; Instituto de Tecnologica Química e Biológica, UNL, Portugal

## Abstract

In this study, several nitrilase genes from phylogenetically distinct organisms were expressed and purified in *E. coli* in order to study their ability to mediate the biotransformation of nitriles. We identified three nitrilases: *Acidovorax facilis* nitrilase (AcN); *Alcaligenes fecalis* nitrilase (AkN); and *Rhodococcus rhodochrous* nitrilase (RkN), which catalyzed iminodiacetonitrile (IDAN) to iminodiacetic acid (IDA). AcN demonstrated 8.8-fold higher activity for IDAN degradation as compared to AkN and RkN. Based on homology modeling and previously described ‘hot spot’ mutations, several AcN mutants were screened for improved activity. One mutant M3 (F168V/L201N/S192F) was identified, which demonstrates a 41% enhancement in the conversion as well as a 2.4-fold higher catalytic efficiency towards IDAN as compared to wild-type AcN.

## Introduction

A major challenge facing the chemical manufacturing industry is the production of bulk chemicals with minimal impact on the environment [1]. Biotransformations, chemical reactions where the traditionally chemical catalyst is replaced by an enzyme, have made a significant impact in addressing this challenge. These reactions are conducted under ambient conditions with a minimal use of toxic reagents [1]. Hydrolases are the most widely employed enzymes in biotransformations, a survey of 134 industrial biotransformations and revealed 44% of those reactions were mediated by hydrolases [2]. Their activities are often independent of expensive cofactors, and substrate specificity can be altered by either natural or laboratory evolution, making them suitable for industrial applications [1], [3]. Identifying new reactions, which could be replaced by biotransformations will further reduce the environmental impact of bulk chemical production.

**Figure 1:**
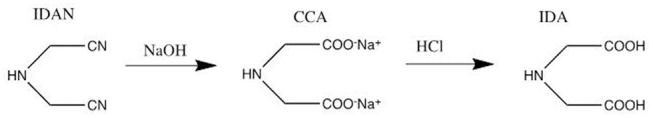
Process for preparation of IDA from IDAN by chemical reaction.

Glyphosate is the most widely used herbicide in the world. Available in over 130 countries, its estimated global production is 600 kilotons, annually [4]. The commercial production of glyphosate requires the synthesis of iminodiacetic acid (IDA) [5], [6]. A general process for IDA production is outlined in Figure 1. Iminodiacetonitrile (IDAN), a dinitrile, is treated with a sodium hydroxide resulting in an IDA sodium salt. The salt is then converted to IDA by the addition of concentrated inorganic acids. This current production process requires the use of strong acid and base, and is estimated to produce 12 tons of wastewater and 1.2 tons of NaCl byproduct per ton of glyphosate [7], [8]. The high-levels of glyphosate production and environmental impact have generated awareness for an alternative production approach [9]–[11]. Enzymatic or greener approaches to perform efficient chemical reactions will be alternative in the production of IDA with several advantages such as mild reaction conditions, environmental friendliness and high activity [8], [10].

Nitrilases (EC 3.5.5.1) are a well-studied class of hydrolases that have been used for several industrial scale biotransformations [12], [13]. These enzymes mediate the hydrolysis of nitriles and dinitriles to their corresponding carboxylic acids [14]. Nitrilases maintain broad substrate specificity and are classified based on their specificity. The majority of nitrilases are specific for aromatic nitriles while others prefer aliphatic or arylacetonitriles substrates [15]–[17]. Based on the promiscuity of nitrilases we sought to investigate these enzymes for their ability to hydrolyze IDAN.

In route we selected an evolutionary diverse set of nine nitrilases for their ability to mediate the biotransformation of IDAN to IDA. The encoding gene was cloned into pET-28b(+) vector and heterologously expressed in *E. coli*. Upon purification the nitrilases were screened for the ability to mediate IDAN biotransformation. Three nitrilases, *Acidovorax facilis* nitrilase (AcN), *Alcaligenes fecalis* nitrilase (AkN), and *Rhodococcus rhodochrous* nitrilase (RkN), demonstrated IDAN hydrolytic activity. AcN demonstrated the highest activity and was further characterized by homology modeling and molecular docking which identified several key residues in IDAN hydrolysis. Mutational analysis identified variant M3 (F168V/L201N/S192F) showed improved activity towards the conversion of IDAN with concentration of 105 mM, as compared to the wild-type AcN.

## Materials and Methods

### Chemicals

T4 DNA ligase and restriction enzymes were purchased from New England Biolabs (Ipswich, MA). DNA polymerase was obtained from Promega (Madison, WI). pET-28b(+) expression vector was purchased from Novagen (Darmstadt, Germany). Iminodiacetonitrile (IDAN) and iminodiacetic acid (IDA) was obtained from Sigma (St. Louis, MO). All other reagents and chemicals were commercially available and of analytic grade.

### Nitrilase Identification

All gene and protein sequences used in this study were obtained from the Protein Data Bank (PDB) and National Center for Biotechnology Information (NCBI). The nitrilase genes from *Acidovorax facilis* (AcN) (GeneBank accession no. DQ444267), *Alcaligene fecalis* ZJUTB10 (AkN) (GeneBank accession no. HQ407378), *Arthrobacter pascens* (ApN) (GeneBank accession no. AB573018), *Burkholderia graminis* C4D1M (BgN) (GeneBank accession no. NZ_ABLD01000011), *Geobacillus pallidus* (GpN) (GeneBank accession no. DQ826045), *Rhodococcus rhodochrous* J1 (RjN) (GeneBank accession no. D11425), *Rhodococcus rhodochrous* K22 (RkN) (GeneBank accession no. D12583), and *Thalassiosira pseudonana* (TpN) (GeneBank accession no. XM_002290007) were synthesized according to the reported methods [18], [19]. DNA manipulation, plasmid isolation, and agarose gel electrophoresis were operated according to standard protocol unless additionally stated.

### Cloning of Nitrilase Genes and Site Directed Mutagenesis

Primers for PCR amplification are listed in Table S1. Reactions were performed on a Thermocycler (Bio-Rad, Hercules, CA) using 20 ng genomic DNA. One PCR cycle consisted of the following: 94°C for 45 s, 55–65°C for 90 s, and 72°C for 3 min. The total cycle number was 35 with a final elongation step at 72°C for 10 min. PCR products were then separated on a 1% agarose gel, purified and then cloned into the pET-28b(+) expression vector. Mutagenesis experiments were performed directly on pET-28b(+)–*AcN* vector according to the published method [20]. The primer pairs designed for mutations are shown in Table S2. One mutagenic PCR cycle consisted of the following: 98°C for 10 s, 55°C for 15 s, and 72°C for 6 min, prior to the mutagenic cycles the reaction was incubated at 94°C for 10 min. Following the PCR, the reactions were treated with 1 U *Dpn*I and incubated for 4 h at 37°C [21]. DNA was purified using QIAquick PCR Purification Kit (Qiagen, Valencia, CA). All pET-28b(+)–*nit* constructs were transformed into *E. coli* BL21 (DE3) by heat shock method [22].

### Enzymes Expression

For enzyme expression, *E. coli* BL21(DE3) cells were selected as the host organism. A single transformed BL21 colony bearing pET-28b(+)–*nit* plasmid was used to inoculate 5 mL of in Lysogeny-Broth (LB) containing 50 µg/mL kanamycin (Kan) and then cultured overnight at 37°C. 1 mL of culture was transferred to 1 L of LB containing 50 µg/mL Kan. The culture was grown at 37°C, 325 rpm until the optical density at 600_nm_ was between 0.6 and 0.8. The culture medium was then supplemented with 0.1 mM isopropyl-*β*-D-thiogalactopyranoside (IPTG), to induce protein expression. Cells were then incubated at 28°C for 20 h and harvested by centrifugation (9,000 rpm, 20 min). Cells were washed twice with 0.9% (w/v) NaCl [23].

### Enzyme Purification

Cell pellets were resuspended in 30 mL 50 mM potassium phosphate (pH 7.5) and lysed by sonication. Lysate was clarified by centrifugation at 9,000 rpm for 20 min at 4°C and the supernatant was retained for purification. The soluble fraction was loaded onto a 10 mL Ni-NTA superflow column pre-equilibrated with 20 mM potassium phosphate, 300 mM sodium chloride (pH 8.0). The column was washed with 20 mM potassium phosphate, 300 mM sodium chloride, and 50 mM imidazole (pH 8.0) to remove any non-specifically bound proteins. The proteins were eluted with 20 mM potassium phosphate, 300 mM sodium chloride, and 500 mM imidazole (pH 8.0). All of these steps are under a constant flow rate of 1 mL/min at 4°C. Protein purification of the eluted fraction was assessed by sodium dodecyl sulfate polyacrylamide (SDS-PAGE) analysis, proteins bands were visualized with Coomassie brilliant blue R-250 [24].

### Circular Dichroism (CD) Measurements

CD spectra were recorded on a JASCO J-815 Spectropolarimeter (JASCO Corporation, Tokyo, Japan) using Spectra Manager 228 software with sensitivity of standard digital integration time (D.I.T) of 2 second, bandwidth of 3.00 nm. Far-UV scans were performed at 0.5 µM protein in 50 mM potassium phosphate (pH 7.5) in a 10-mm cuvette. The spectra were recorded from 200 nm to 250 nm with a scan speed of 100 nm/min at 25°C. Data were expressed as mean residue ellipticity ([θ]_mrw,λ_) (in deg•cm^2^•dmol^−1^) as described previously [25]. Thermal denaturation of enzymes was followed as a function of temperature by continuously monitoring ellipticity changes at 222 nm using a step size of 0.4°C. The melting temperature (T_m_) was calculated by taking the first order derivative of the sigmoidal curve obtained from the melting curve [26].

### Nitrilase Activity Assay

It is reported that the adiponitrile (ADPN) is suitable for nitrilase screening and activity assay [16], [17], in this study, nitrilase activity was determined by the hydrolysis of ADPN. Reactions were performed in 10 mL of buffer (50 mM citrate (pH 3.0, 4.0, 5.0), 50 mM potassium phosphate (pH 6.0, 7.0), 50 mM Tris-HCl (pH 8.0), 50 mM glycine-sodium hydroxide (pH 9.0, 10.0) containing 50 mM ADPN at 35°C. These reactions were carried out at temperatures ranging from 20 to 70°C with a reciprocal shaker at 150 r/min. ADPN was assayed using Agilent 6890N gas chromatograph (Agilent, Santa Clara, CA) equipped with a flame ionization detector (FID) and a FFAP column (30 m×0.25 mm×0.33 µm). The operating conditions were as follows: temperature of oven 180°C, the temperatures of injection and detector kept at 250°C. The retention time for ADPN is 2.9 min. One unit of the enzyme activity toward ADPN was defined as the amount of enzyme (mg protein) required to reduce 1 µmol of ADPN per minute at 35°C. Protein quantitative analysis was determined by the Bradford method with bovine serum albumin as a standard [27]. All reactions were performed in triplicates.

### IDAN Hydrolysis Kinetics

*K*_cat_ and *K*_m_ values were determined from initial-velocity data measured as a function of substrate concentration. Enzyme reactions were carried out at 35°C. Prior to the reaction, the enzymes were incubated at 35°C for 5 min. The reaction was initiated by addition of purified enzyme (0.1 g/L) to the different concentration of substrate solutions (10.5–262.5 mM of IDAN) in 10 mL of 50 mM potassium phosphate (pH 7.5) in an Erlenmeyer flask on a rotary shaker at 150 rpm. The reaction mixture was incubated at 35°C, and 1 mL aliquots were taken every 30 min and the reaction was stopped with the addition of 100 µL of 1 M H_2_SO_4_. 20 µL of reaction mixture was directly applied onto the high-performance liquid chromatography (HPLC) for analysis. Hypersil SAX ion exchange column (Shimadzu, Kyoto, Japan) was used for quantitative analysis of IDA, 2-((cyanomethyl)amino) acetic acid (CCA), and IDAN. The parameters used for the detection of the compounds were a UV detector set at a wavelength of 210 nm, a flow rate of 1.0 mL/min, and a mobile phase of 20 mM ammonium phosphate with pH 4.0. The retention times for IDAN, CCA and IDA were 3.2, 4.2 and 8.1 min, respectively. Peak areas were quantified using specific external standards, and the absolute configuration was identified by comparing the HPLC retention times with those of standard samples. The initial velocity data obtained were fitted to the equation *v* = *V*_max_[S]/([S]+*K*_m_) in which *v* is the initial velocity, *V*_max_ is the maximum velocity, [S] is the substrate concentration, and *K*_m_ is the Michaelis constant, respectively, by using Origin software (OriginLab Corporation, Northampton, MA). The *K*_cat_ was calculated from the ratio of *V*_max_ to enzyme concentration. One unit of the enzyme activity toward IDAN was defined as the amount of enzyme required to produce 1 µmol of IDA per minute at 35°C. Protein quantitative analysis was determined by the Bradford method with bovine serum albumin as the standard [27]. All reactions were performed in triplicate.

### Time Courses of Iminodiacetonitrile Hydrolysis

To assess the production of IDA and the intermediate CCA using recombinant wild type nitrilase and mutant M3, time courses of IDAN hydrolysis were examined. The purified proteins were diluted to 0.1 g/L in 50 mM potassium phosphate (pH 7.5). The reactions were initiated with the addition of 105 mM IDAN. Samples were removed at predetermined times and the concentrations of IDAN, CCA and IDA were determined as described above.

### Homology Modeling and Docking

Nitrilases models were generated using Build Homology Models (MODELER) in Discovery Studio 2.1 (DS 2.1) (Accelrys Software, San Diego, CA). Templates for structures modeling were selected according to the sequences similarity. Models were constructed based on the crystal structures of *Pyrococcus abyssi* nitrilase (PDB accession code 3IVZ), hypothetical protein from *Pyrococcus horikoshii* (PDB accession code 1J31), AmiF formamidase from *Helicobacter pylori* (PDB accession code 2E2L), and *Mus musculus* nitrilase (PDB accession code 2W1V). Generated structures were improved by subsequent refinement of the loop conformations by assessing the compatibility of an amino acid sequence to known PDB structures using the Protein Health module in DS 2.1. The geometry of loop regions was corrected using Refine Loop/MODELER. The best quality model was chosen for further calculations, molecule modeling, and docking studies by Autodock 4.0 [28]. Sequence alignments were performed using the program ClustalX [29]. Charge distribution over the entire molecule surface was calculated using the Adaptive Poisson-Boltzmann Solver software [30], and the rendering of the 3D-structure and aligning were using the PyMol ver 0.99 (Schrodinger, Portland, OR).

## Results

### Nitrilases Identification, Expression and Purification

**Figure 2:**
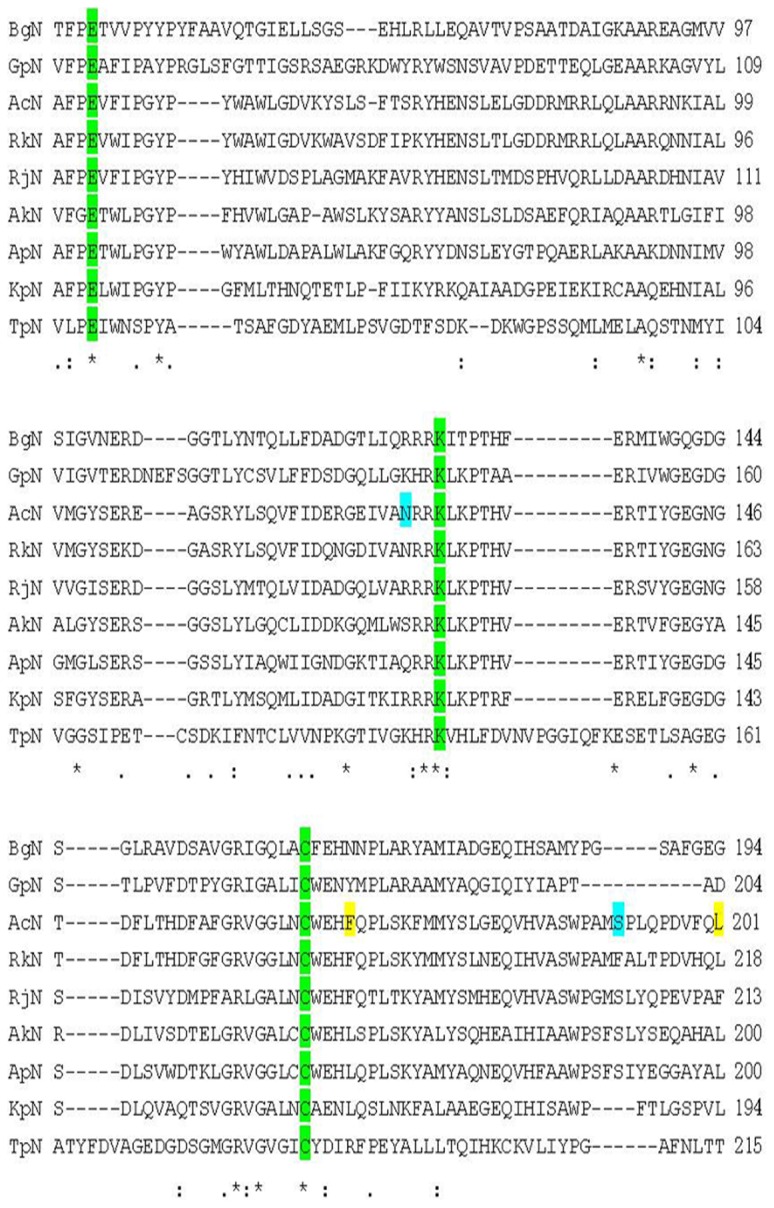
Multiple sequence alignment of nitrilase sequences. Highlighted in green are catalytic residues, previously described mutations in yellow (41, 42), and mutations identified in this study are in blue. ‘*’ denotes residues that are identical in all sequences, ‘:’ denotes conserved substations and ‘.’ indicates semi-conserved substitutions.

To identify a diverse set of nitrilase sequences, a BLAST search was performed using AkN, a well-described nitrilases, as the template sequence [20]. Sequences that demonstrated <60% homology were selected. A multiple sequence alignment was constructed with nitrilase sequences from *Acidovorax facilis*, *Alcaligene fecalis*, *Arthrobacter pascens*, *Burkholderia graminis*, *Geobacillus pallidus*, *Rhodococcus rhodochrous*, *Rhodococcus rhodochrous*, and *Thalassiosira pseudonana* using *Pyrococcus abyssi* nitrilase (PaN) (PDB code 3KLC), a well-characterized nitrilase with a known crystal structure, as the template sequence [31] (Figure 2). Catalytic triad residues of PaN (E120, K278, and C329) were conserved in all nine nitrilase sequences. The percent identity was calculated to determine the sequence diversity of this set of nitrilases (Table S3). Compared to PaN, the percentage of identity ranged from as low as 13.7% for BgN to as high as 20.6% for RkN. ApN and AkN demonstrated 14.1 and 14.5% sequence identity to PaN. TpN and GpN displayed a higher sequence identity 15.6 and 17.1%, respectively. KpN and RjN similarly displayed 17.9% sequence identity to PaN. AcN showed an 18.7% sequence identity to PaN. The nine nitrilases were recombinantly expressed in the bacterial host *E. coli* BL21 (DE3) cells and purified using immobilized-metal affinity chromatography. All the purified enzymes were analyzed by sodium dodecyl sulfate polyacrylamide gel electrophoresis (SDS-PAGE) (Figure S1). The apparent molecular weights of the proteins ranging from 32 to 44 kDa, was in agreement with the expected molecular weights from DNA sequences (Table S4).

### Secondary Structure Analysis

CD studies were performed to assess the conformational integrity of these nitrilases. All nitrilases exhibited far ultraviolet CD spectra, which exhibited a double minimum at 208 and 222 nm, indicating they were all α/β proteins (Figure S2) [32]. To compare the stability of the proteins, the unfolding of the protein was then monitored by the change in ellipticity at 222 nm as the temperature of the sample increased (Figure S3). All transitions were found to be cooperative and irreversible and had thermal stabilities with T_m_ of 46.8 to 57.2°C (Table S4). This data suggests that these nitrilases maintain their conformation under mild conditions, suggesting their candidacy for biotransformations.

### Optimization of ADPN Hydrolysis

**Figure 3:**
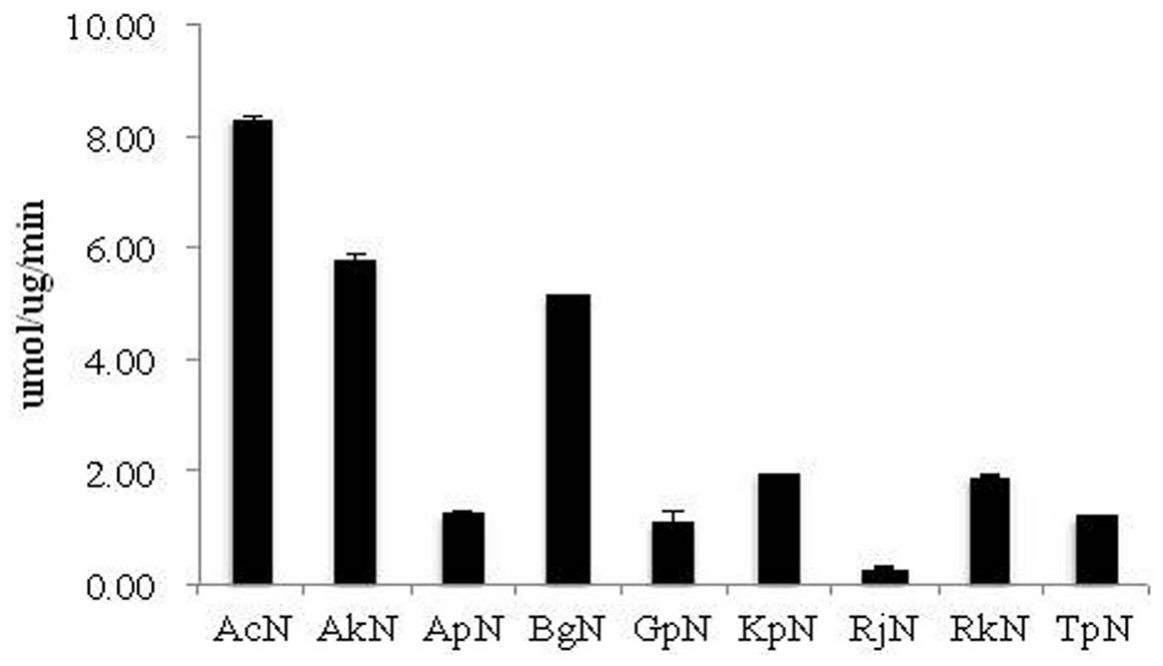
Activity assay for ADPN hydrolysis. The reactions were performed at 35°C 150 rpm, for 30 min. 0.1 g/L purified nitrilase and 50 mM ADPN were added to 10 mL potassium phosphate (50 mM, pH 7.5). Error bars represent the standard deviation from three separate trials.

The ability of nitrilases to hydrolyze ADPN was examined. All nitrilases demonstrated ADPN hydrolysis activity (Figure 3). AcN demonstrated the highest activity for ADPN, 8.29±0.05 µmol/mg/min. AkN and BgN also displayed high activity, 5.80±0.1 and 5.14±0.04 µmol/mg/min, respectively. Modest activity was detected for KpN (1.97±0.02 µmol/mg/min) and RkN (1.94±0.01 µmol/mg/min). The remaining nitrilases ApN, TpN, GpN, and TpN all demonstrated low but significant ADPN hydrolytic activity, 1.26±0.05, 1.22±0.02, 1.13±0.17, and RjN 0.28±0.01 µmol/mg/min, respectively. Thus, ADPN can be used as a suitable substrate to determine the optimal reaction conditions of these enzymes. The effects of pH and temperature on each enzyme activity for substrate ADPN were assessed. AcN exhibited maximum activity at pH 7.0 (Figure S4). The optimal temperature was 40°C, and enzyme activity was rapidly lost above 60°C (Figure S5). Optimal activity of AkN, ApN, BgN RjN and RkN was observed at pH 8.0. GpN, KpN and TpN demonstrated optimal activity at pH 7.0. AcN, AkN, ApN, RjN and TpN were tolerant to acidic conditions. These enzymes maintained greater than 50% of their activity at pH 5.0. Under extreme acidic conditions (pH 4.0) all enzymes experienced a significant loss of activity (greater than 60%). All the nitrilases investigated displayed less than 50% of their optimal activity under alkaline conditions. The temperature-dependent studies of nitrilase hydrolysis showed that AkN, ApN, BgN, KpN, and RjN all had optimal temperatures at 40°C (Figure S5). GpN, RkN and TpN had slightly higher optimal activities at 50°C. At 60°C AcN, GpN and KpN retained more than 50% of their activity at 40°C. AkN, ApN, BgN, RjN, RkN, and TpN demonstrated less thermal stability with a greater than 50% loss in activity at 60°C. At 70°C all nitrilases investigated in this study demonstrated less than 10% activity.

### IDAN Activity

**Figure 4:**
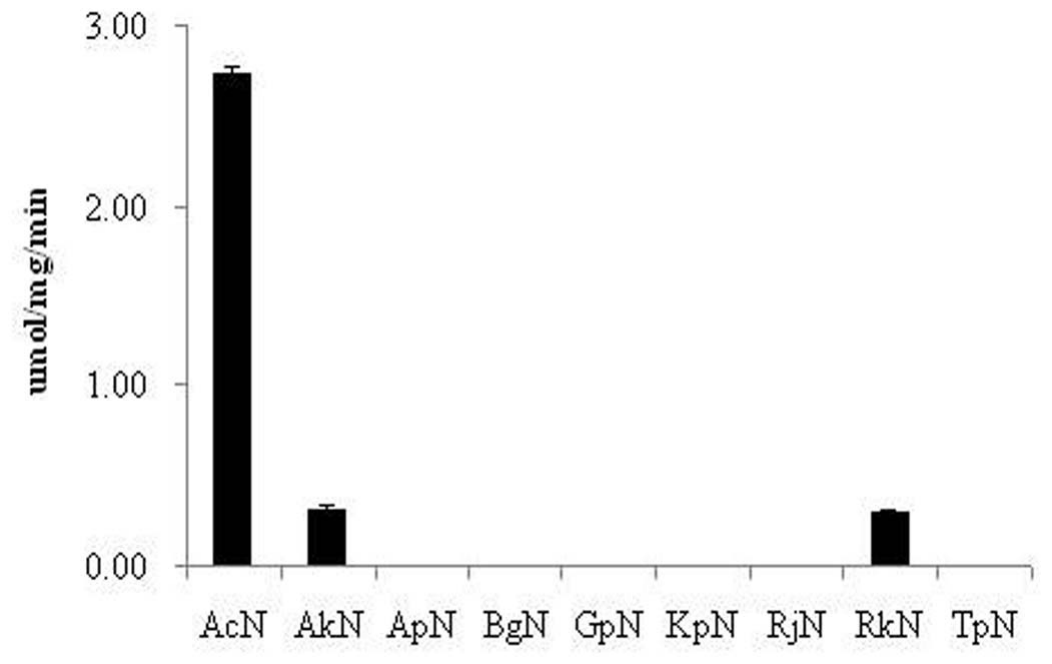
Comparison of wild-type nitrilases for IDAN hydrolytic activity in 50 mM potassium phosphate (pH 7.5) at 35°C for 2 h. The concentration of IDAN was 105 mM. The activity was assayed according to the standard methods. Error bars represent the standard deviation from three separate trials.

IDAN activity was assessed to identify nitrilase sequences that were active towards this substrate. Reactions were performed in 10 mL 50 mM potassium phosphate (pH 7.5) containing 0.1 g/L purified nitrilase at 35°C, reactions were initiated upon the addition of 105 mM IDAN. HPLC reference peaks for IDAN, CCA, and IDA were established at 3.2, 4.2, and 8.1 min, respectively. After 2 hours the reaction mixtures were subjected to HPLC and compared to reference peaks. Chromatograms of ApN, BgN, GpN, RjN, and TpN reactions demonstrated a single peak with a retention time of 3.2 min, suggestive of inactivity towards IDAN (Figure S6). Three peaks were observed in the chromatograms of AcN, AkN and RkN, corresponding to the each of the reference peaks (Figure S7 in File S1). Integration of the IDA peak demonstrated AcN had the highest activity, 2.73±0.03 µmol/mg/min. 8.8-fold lower activity is reported for AkN and RkN, 0.31±0.01 and 0.30±0.01 µmol/mg/min, respectively (Figure 4). Based on these results we further characterized AcN.

### Protein Homology Modeling and Docking Analysis

**Figure 5:**
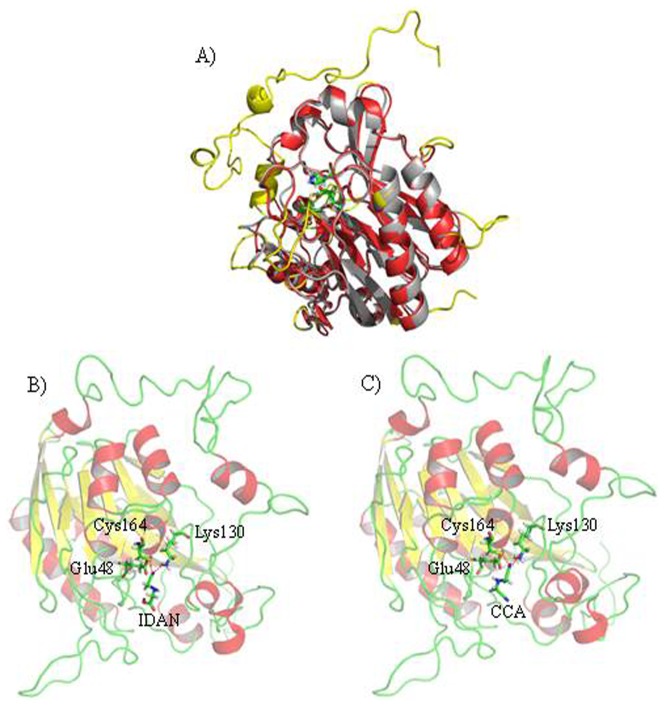
Structural analysis of AcN A) overlay of AcN (grey) overlay of PaN (red) with active site residues rendered as stick revealing nearly identical structural similarity. The non-overlapping regions are highlighted in yellow. B) Docking analysis of AcN with IDAN, dashed lines represent H-bonds (red), carbon atoms (green), hydrogen atoms (grey), nitrogen atoms (blue), oxygen atoms (red), and sulfur atoms (orange). C) Docking analysis of AcN with CCA, dashed lines represent H-bonds (red), carbon atoms (green), hydrogen atoms (grey), nitrogen atoms (blue), oxygen atoms (red), and sulfur atoms (orange).

**Figure 6:**
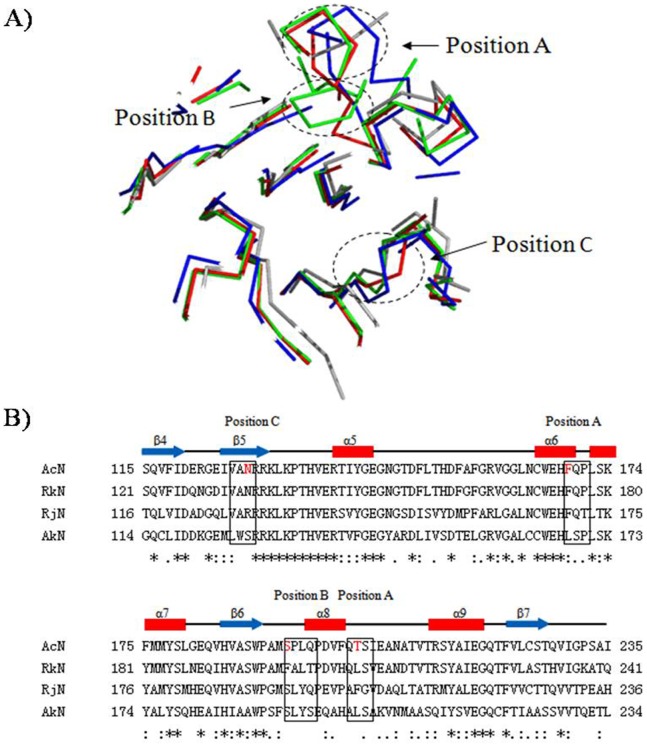
Mutations selection based on the primary and secondary structures of nitrilases. A. Superimpose of four nitrilases catalytic trid pocket (CEK) expand 12 Å area with ribbon style. red: AkN green: RjN blue: RkN gray: AcN. Distinctly different conformations displayed in the A B C ellipses. B. Alignment of four nitrilases RkN, RjN, AkN and AcN. Residues identical in all sequences in the alignment are labeled with *, Residues with conserved substations are labeled with :, and with. for semi-conserved substitutions.

Homology modeling of these nitrilases was performed to determine the active site conformation of these proteins (Figure S8). Models were generated from *Pyrococcus abyssi* nitrilase (PDB accession code 3IVZ), hypothetical protein from *Pyrococcus horikoshii* (PDB accession code 1J31) [31], [33], AmiF formamidase from *Helicobacter pylori* (PDB accession code 2E2L), and *Mus musculus* nitrilase (PDB accession code 2W1V) [34]. Comparative modeling was used to generate the most probable structure of the AcN by the alignment with template sequences, while simultaneously satisfying spatial restraints and local molecular geometry **(** Figure 5A **)**. Despite varying sequence identity, all nitrilase models demonstrated a characteristic monomer fold and the E, K, and C residues of the catalytic triad presented similar geometry (Figure S9). Finally, the best quality models were chosen for further calculations, molecular modeling, and docking studies. These studies were performed to demonstrate the *in silico* interactions between the enzyme and IDAN. A 60 Å^3^ area around the catalytic triad pocket was defined as the active site [31], [34]. The docking of IDAN to the active site of AcN **(** Figure 5B **)**, indicated a hydrogen bond (bond length 2.2 Å) between N_1_ of IDAN and the -SH moiety group of C164. A second hydrogen bond (1.8 Å) was identified between N_1_ of IDAN and K130. Docking experiments were also performed with the CCA intermediate **(** Figure 5C **)**. This data revealed a hydrogen bond between CCA and C164 (2.5 Å). A second hydrogen bond was observed between the nitrogen atom of CCA and K130 (2.4 Å). These results demonstrate that geometry of the AcN active site can accommodate both IDAN and CCA. To identify structural features which affect IDAN activity, the AcN model was superimposed on models representing other nitrilase families: aromatic nitrilase (RjN) [35], aliphatic nitrilase (RkN) [33], and arylacetonitrilase (AkN) (Table S6) [36], Positions A/B/C displayed distinct structural conformations in AcN as compared to the other nitrilases (Figure 6A). Sequence analysis of these regions showed several non-conserved and semi-conserved substitutions in these regions (Figure 6B). This data is suggestive that residues in A/B/C position may influence the substrate specify of these enzymes.

### Kinetic Analysis of Wt-AcN and Mutants

Based on the homology models (Figure 5 and Figure 6A), sequence alignments (Figure 2 and Figure 6B) and previously reported data, the following AcN mutants were generated [F168V, L201N, N127R, S192F, S192H, C164F and M1 (F168V/L201N), M2 (N127R/C164F), M3 (F168V/L201N/S192F)] [37], [38] to improve the AcN activity on IDAN. IDAN activity of these mutants was compared to wild-type AcN (WT-AcN) (Table 1). Wt-AcN exhibited a *k_cat_* of 3.25±0.13 s^−1^, a K_m_ of 0.76±0.02 mM^−1^ and an overall catalytic efficiency (*k_cat_*/K*_m_*) of 4.30±0.08 s^−1^ mM^−1^. Mutants N127R, C164F, and M2, demonstrated no activity towards IDAN. Relative to Wt-AcN all active mutants demonstrated increased *k_cat_* values. S192H was marked with a 1.52-fold increase as compared to Wt-AcN, 4.93±0.39 s^−1^. The *k_cat_* of F168V was 5.04±0.37 s^−1^, a 1.55-fold improvement over Wt-AcN. A 1.61-fold increased *k_cat_* was recorded for L201N, 5.23±0.14 s^−1^. S192F displayed a *k_cat_* of 5.26±0.08 s^−1^. Mutants M1 and M3 showed the most improvement as compared to Wt-AcN, 5.52±0.21 and 5.69±0.19 s^−1^, respectively. The mutants that demonstrated improved *k_cat_* values also showed a decrease in K*_m_*. The K*_m_* for L201N, S192F, and S192H were 0.64±0.06, 0.63±0.03, and 0.62±0.04 mM^−1^, respectively. M1 and M3 mutants demonstrated K*_m_* values of 0.56±0.03 and 0.55±0.04 mM^−1^ representing a 1.36- and 1.38-fold decreases in K*_m_* as compared to Wt-AcN. In terms of catalytic efficiency mutants F168V, L201N, S192F, S192H, M1, and M3, all out performed Wt-AcN. S192H, L201N, S192F and F168V, demonstrated a 1.84-, 1.92-, 1.94-, and 2.08-fold increase in catalytic efficiency as compared to Wt-AcN. The most dramatic increase in catalytic efficiencies was observed for M1 and M3, 9.86±0.19 and 10.44±0.53 mM^−1^, respectively. After mutations, the conversion of mutants of IDAN improved, the highest conversion was observed for M3 with conversion of 96%, and 1.48-fold increase as compared to Wt-AcN (conversion of 65%).

**Table 1:** Kinetic analysis of WT-AcN and mutants for IDAN hydrolysis at 35°C, pH 7.5.

can	*k_cat_* s^−1^	*K_m_* mM^−1^	*k_cat_*/*K_m_* mM^−1^s^−1^	Conversion (%)
WT	3.25±0.13	0.76±0.02	4.30±0.08	65
F168V	5.04±0.37	0.57±0.03	8.94±0.25	80
L201N	5.23±0.14	0.64±0.06	8.26±0.91	82
N127R	ND	ND	ND	ND
S192F	5.26±0.08	0.63±0.03	8.36±0.35	86
S192H	4.93±0.39	0.62±0.04	7.90±0.21	73
C164F	ND	ND	ND	ND
M1(F168V/L201N)	5.52±0.21	0.56±0.03	9.86±0.19	89
M2(N127R/C164F)	ND	ND	ND	ND
M3(F168V/L201N/S192F)	5.69±0.19	0.55±0.04	10.44±0.53	96

Note: ND, not detectable.

### Time Course for Quantitative Analysis of IDAN Biotransformation

**Figure 7:**
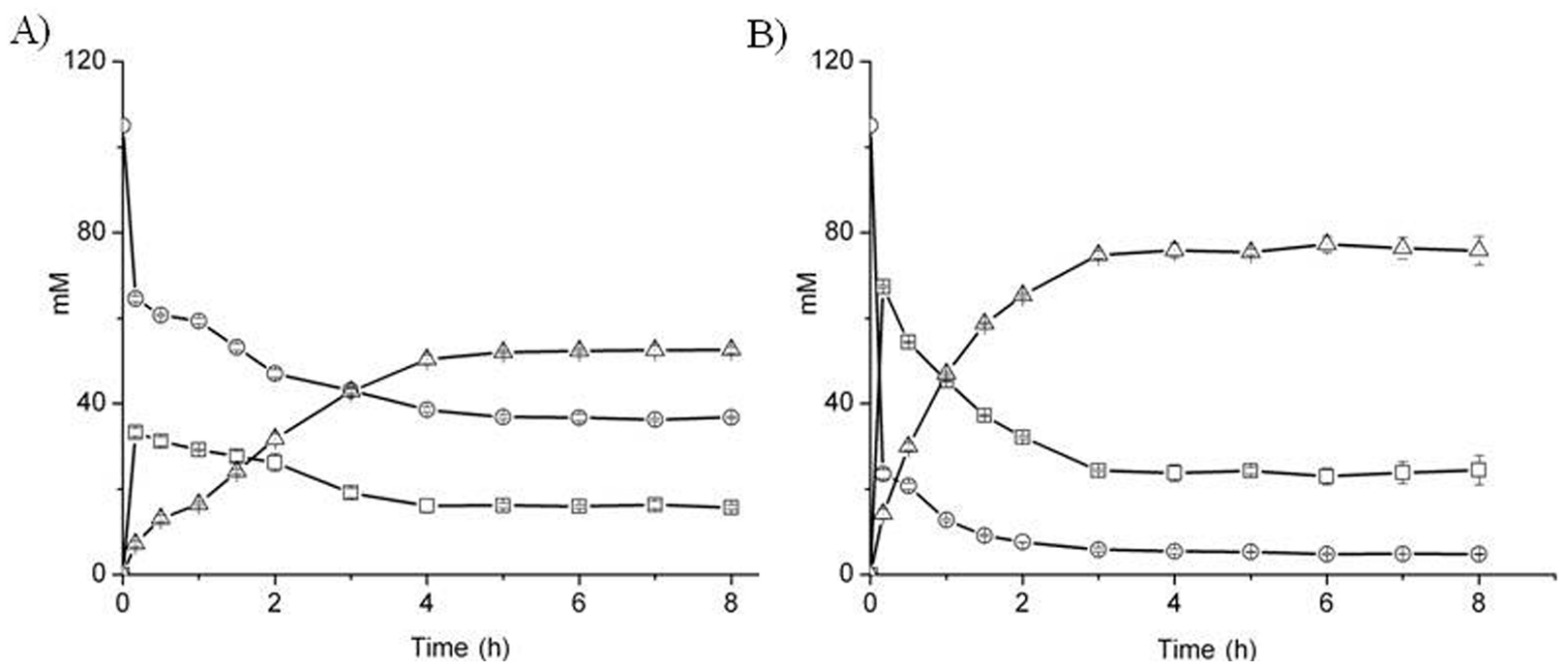
Time course analysis of IDAN biotransformation by (A) AcN and (B) M3 under optimal conditions with pH of 7.5, temperature of 35°C and concentration of IDAN of 105 mM, (open circles) IDAN, (open squares) CCA, and (open triangles) IDA.

The time course for Wt-AcN and the M3 production of CCA and IDA from 105 mM IDAN is shown in Table 2. Both enzymes were able to convert IDAN to CCA and then CCA to IDA, although their rates differ considerably. After 0.5 hours, Wt-AcN converted 42% of IDAN resulting in yield of 70% CCA and 30% IDA, whereas M3 converted 80% of IDAN yielding 62% CCA and 38% IDA. At subsequent time points this trend, where M3 demonstrated increased IDAN conversion and increased percent yield of IDA, continued. The Wt-AcN biotransformation plateaued after 4 hours and for M3 this plateau occurred after only 3 hours (Figure 7). The final yields from the Wt-AcN reactions were 65% of IDAN was converted and 15.73±0.59 mM of CCA and 52.53±0.78 mM of IDA were generated. In contrast, M3 converted 96% of IDAN resulting in 22.97±1.93 mM of CCA and 77.26±1.86 mM of IDA. These results further confirmed that after mutations, the mutant M3 showed better catalytic efficiency on the IDAN as compared to wild type AcN.

**Table 2:** Comparison of CCA and IDA production from IDAN by Wt-AcN and mutant M3 at different time points.

	0.5 h (mM)			1.0 h (mM)			2.0 h (mM)		
	IDAN	CCA	IDA	IDAN	CCA	IDA	IDAN	CCA	IDA
WT	60.74±0.3	31.17±1.02	13.13±0.72	59.31±0.63	29.20±0.20	16.53±0.44	47.04±0.93	26.23±2.10	31.77±1.16
M3	20.73±0.75	50.37±0.15	29.94±0.85	12.78±0.36	45.29±0.12	46.97±0.39	7.61±0.04	32.15±0.38	65.29±0.42

## Discussion

Industrial enzymes such as nitrilase offer significant improvements over traditional chemical catalysts in process chemistry [39]. Nitrilases have unparalleled advantages in terms of efficiency, selectivity, optical purity, and more environmental friendly reaction conditions [40]. Despite these advantages, the current repertoire of nitrilases is limited in the synthesis of di-acids including adipic acid and IDA due to product inhibition [41], [42]. The goal of these studies is to identify and characterize new nitrilase sequences that can be used in IDA production.

In the present study, the hydrolysis of ADPN and IDAN to adipic acid and IDA by nitrilases has been demonstrated. However, of the nine evolutionary distinct nitrilase sequences identified and screened for ADPN and IDAN activity, only AcN, AkN and RkN were active for both substrates (Figure 3 and 4). Previously, we have reported on the AkN-mediated production of IDA by either whole cell synthesis or with the use of immobilized enzymes [8], [10]. Whole cell production of IDA was limited due to poor substrate recognition and sensitivity to environmental factors such as temperature and pH [10]. Although modest improvements in stability were observed upon immobilization, overall production was limited due to the low specific activity [8]. Without a detailed crystal structure of AkN or a high-throughput screen for IDA production, rational design or laboratory evolution were avoided. We sought an *in silico* approach which would take advantage of both the naturally occurring nitrilase sequence diversity and previously described nitrilases mutations.

The sequence diversity of nitrilases has been well described in the literatures [43], [44]. Robertson *et al.* screened 137 unique nitrilases sequences and mapped their activities to their position on the phylogenetic tree. These results suggested that evolution has influenced the active site of nitrilases to accommodate and hydrolyze structurally distinct substrates [44]. Based on our selection criteria nine nitrilases, which represented distinct evolutionary lineages, were selected (Figure S10). Of these nine sequences, only three (AcN, AkN, and RkN) demonstrated activity (Figure 4) on IDAN. AcN showed a dramatic increase in specific activity on IDAN as compared to other nitrilases examined in this study.

Wild-type and recombinant AcN mediate the biotransformations of 3-hydroxynitriles (3-HVN) to 3-hydroxycarboxylic acids (3-HVA) and glycolonitrile (GLN) to glycolic acid (GLA) [45]–[47]. Previous reports demonstrated that AcN was active toward xenobiotic substrates and the activity was amenable to mutations near the activity site, saturation mutagenesis experiment active site have identified several positions, which influence these biotransformations [37], [38]. Individually, F168V and L201N mutants demonstrated a 4.1- and 5.5-fold increase in specific activity for GLN. These mutations also function synergistically where the double mutant F168V/L201N demonstrated a 15.3-fold increase in specific activity as compared to the wild type enzyme [37]. We demonstrated that this synergistic effect is also observed for IDAN where the catalytic efficiency (*k_cat_*/K*_m_*) of M1 (F168V/L201N) demonstrated a 2.3-fold improvement over the wild-type (Table 2).

Docking experiments identified S192, located on the flexible loop in the binding cleft, as another potentially important region for altering enzyme activity. Replacing S192 with bulkier H and F residues decreased the size substrate-binding pocket and resulted in 1.8 and 1.9-fold improvement in *k_cat_*/K*_m_,* respectively (Figure 7 and Table 2). Combining the previously described F168V/L201N mutations with the S192F we generated the M3 mutant. The M3 mutant demonstrated a significant improvement over Wt-AcN 2.4-fold improvement in *k_cat_*/K*_m_*, and conversion towards IDAN from 65% to 96%.

### Conclusions

In summary, nine recombinant nitrilases from genetically distinct backgrounds were constructed and investigated for nitriles hydrolysis. Among these nitrilases three were able to mediate the biotransformation of IDAN. In particular, AcN demonstrated significant hydrolytic activity when compared to the other species. Mutations were selected based on the homology modeling and previous studies to improve the activity of the AcN for IDAN hydrolysis. The M3 mutant identified in this study demonstrated that the ability of this mutant to catalyze the IDA production was improved and laid the foundation for the production of IDA on the industrial scale.

## Supporting Information

Figure S1 **SDS-PAGE analysis of purified nitrilases**. 1) BgN 2) AkN 3) TpN 4) RkN 5) GpN M) molecular weight marker 6) AcN 7) KpN 8) ApN and 9) RjN.
(TIF)

Figure S2 **CD wavelength scans of 1) AcN 2) AkN 3) ApN 4) BgN 5) GpN 6) KpN 7) RjN 8) RkN 9) TpN.** All scans were performed at 30°C in 50 mM potassium phosphate buffer (pH 7.5).
(TIF)

Figure S3 **CD temperature profiles of 1) AcN 2) AkN 3) ApN 4) BgN 5) GpN 6) KpN 7) RjN 8) RkN 9) TpN at 222 nm.** All scans were performed in 50 mM potassium phosphate buffer (pH 7.5).
(TIF)

Figure S4 **pH activity profile of 1) AcN 2) AkN 3) ApN 4) BgN 5) GpN 6) KpN 7) RjN 8) RkN 9) TpN.** Data is normalized to activity at pH 7.0 for each enzyme. Error bars represent the standard deviation from three separate trials.
(TIF)

Figure S5 **Temperature profile of 1) AcN 2) AkN 3) ApN 4) BgN 5) GpN 6) KpN 7) RjN 8) RkN 9) TpN for IV.** Data is normalized to activity at 40°C. Error bars represent the standard deviation from three separate trials.
(TIF)

Figure S6 **HPLC spectrums of nitrilases which demonstrated no activity for IDAN hydrolysis assay.** 1) ApN 2) BgN 3) GpN 4) KpN 5) RjN and 6) TpN. The retention times for IDAN, CCA and IDA peaks were 3.4, 4.2, and 8.1 minutes, respectively.
(TIF)

Figure S7 **HPLC spectrums of nitrilases which demonstrate IDAN hydrolysis activity.** 1) AcN, 2) AkN and 3) RkN. The retention times for IDAN, CCA and IDA peaks were 3.4, 4.2, and 8.1 minutes, respectively.
(TIF)

Figure S8 **Homology protein models of nitrilases.** A) AcN B) ApN C) BgN D) GpN E) RjN F) AkN G) RkN H) KpN and I) TpN. Helix, sheet, loop are displayed in red, yellow and green, respectively.
(TIF)

Figure S9 **Alignment of nitrilase catalytic triads.** AcN (red), AkN (green), ApN (blue), BgN (green), GpN (pink), KpN (purple), RjN (light blue), RkN (black) and TpN (orange).
(TIF)

Figure S10 **Phylogenetic tree for the nitirlases used in this study based on the sequences identity.**
(TIF)

Table S1 **Primers used for PCR amplification of nitrilase genes.**
(DOC)

Table S2 **Primers used for site directed mutagenesis of AcN mutants.**
(DOC)

Table S3 **Comparison similarity of nitrilases with different protein sequences.**
(DOC)

Table S4 **The expected and experimental molecular weights of nine nitrilases.**
(DOC)

Table S5 **Melting temperatures of nitrilases used in this study as determined by CD.**
(DOC)

Table S6 **Docking analysis of AcN, AfN and RkN with IDAN.**
(DOC)
