# Supplementary figures and images for: Screening and Improving the Recombinant Nitrilases and Application in Biotransformation of Iminodiacetonitrile to Iminodiacetic Acid
Source: PLoS One. 2013 Jun 27;8(6):e67197. doi: 10.1371/journal.pone.0067197 (PMC3695085; doi:10.1371/journal.pone.0067197)

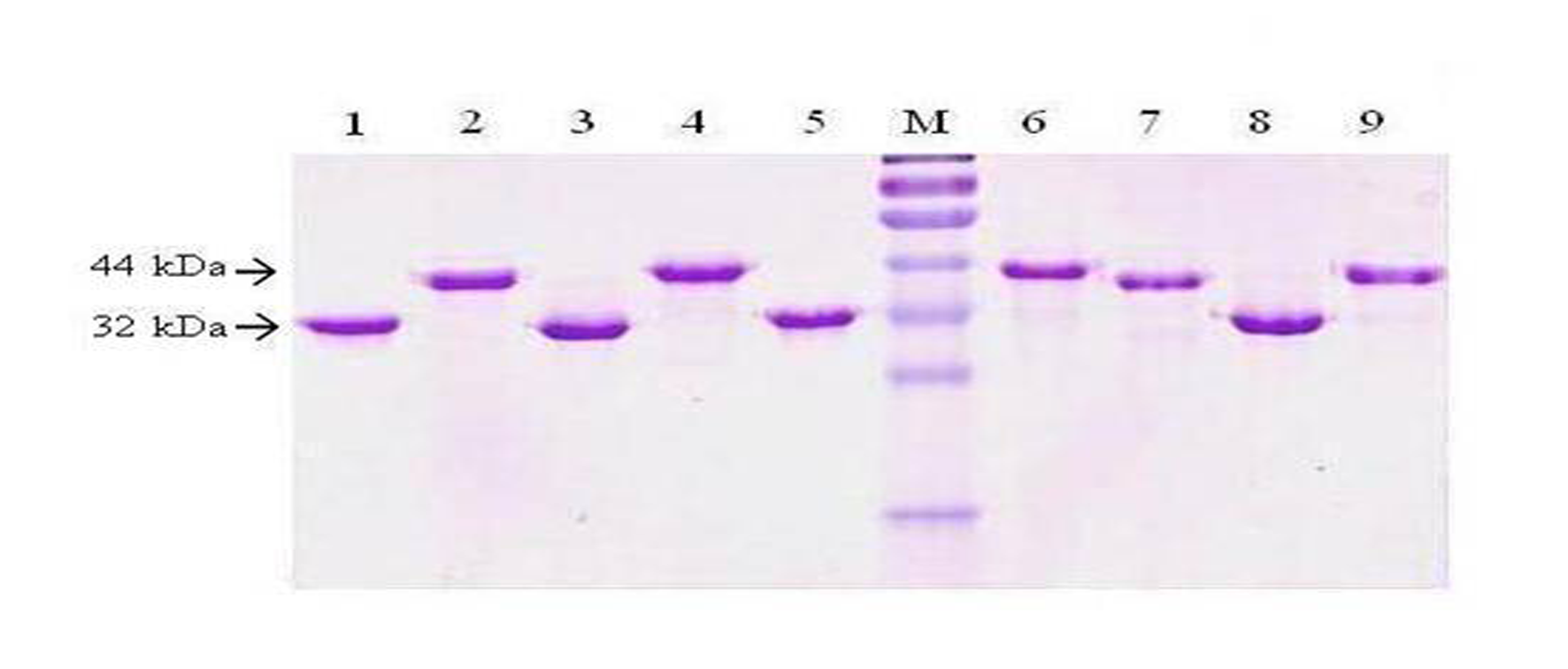

Supplement: Figure S1 — SDS-PAGE analysis of purified nitrilases. 1) BgN 2) AkN 3) TpN 4) RkN 5) GpN M) molecular weight marker 6) AcN 7) KpN 8) ApN and 9) RjN. (TIF) [file pone.0067197.s001.tif]

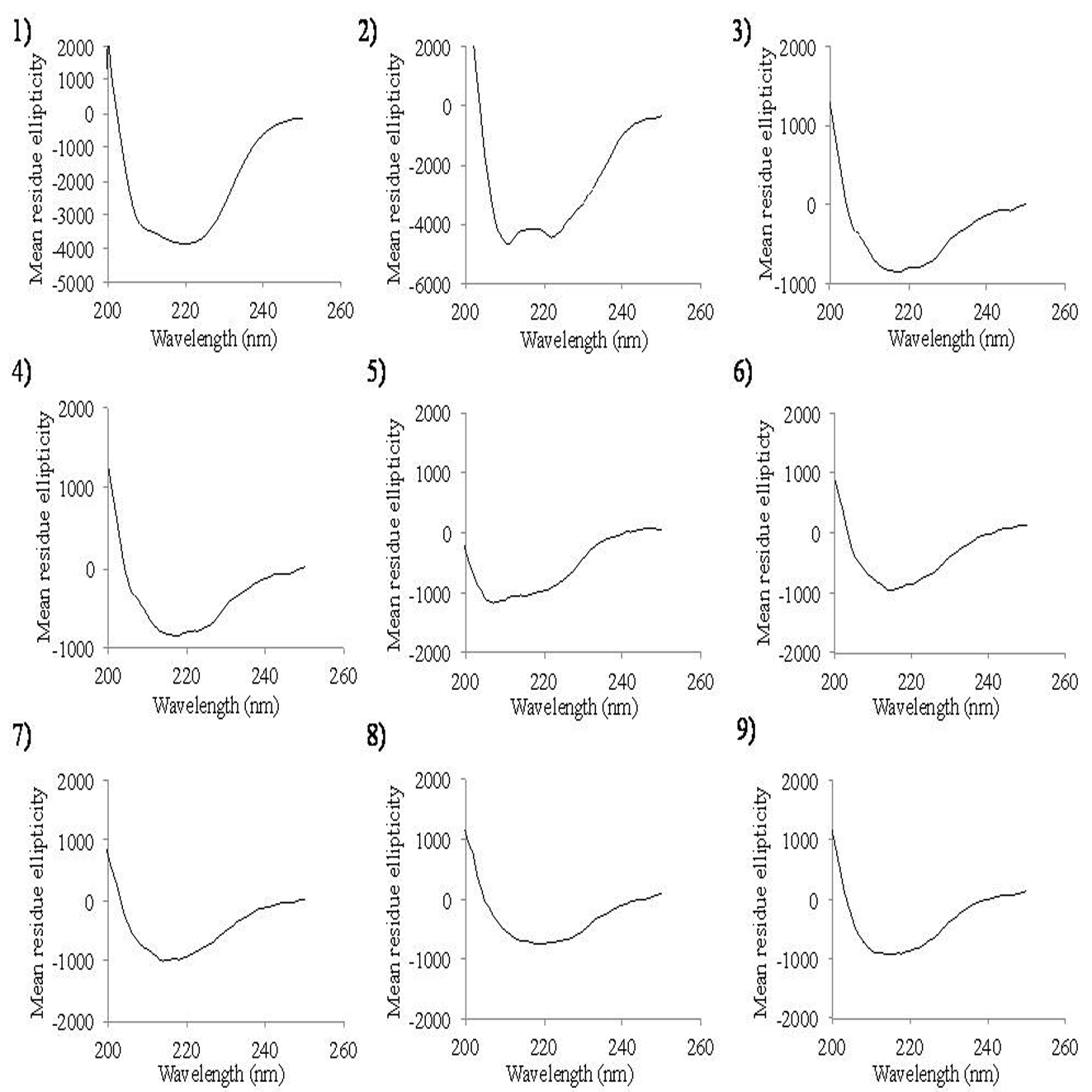

Supplement: Figure S2 — CD wavelength scans of 1) AcN 2) AkN 3) ApN 4) BgN 5) GpN 6) KpN 7) RjN 8) RkN 9) TpN. All scans were performed at 30°C in 50 mM potassium phosphate buffer (pH 7.5). (TIF) [file pone.0067197.s002.tif]

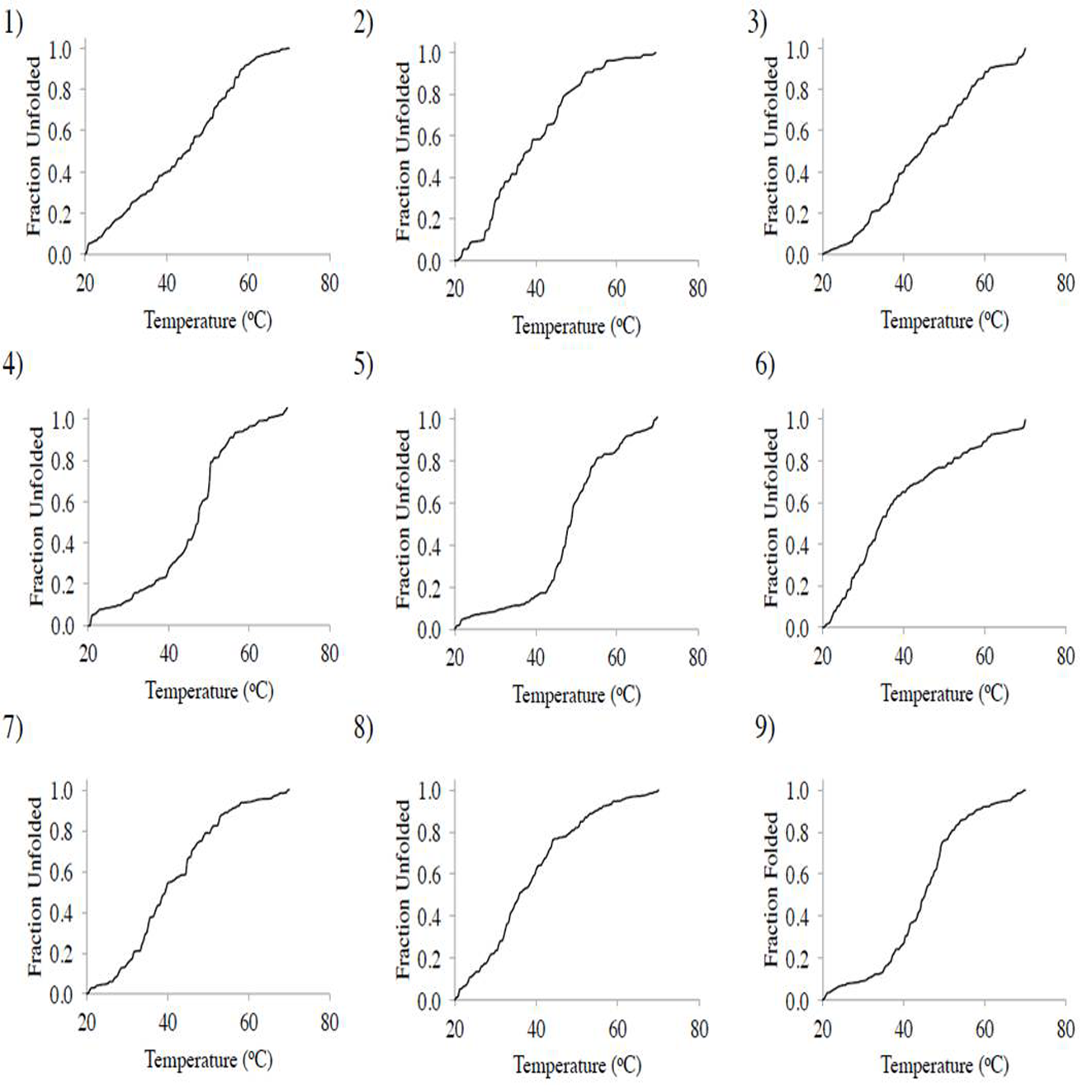

Supplement: Figure S3 — CD temperature profiles of 1) AcN 2) AkN 3) ApN 4) BgN 5) GpN 6) KpN 7) RjN 8) RkN 9) TpN at 222 nm. All scans were performed in 50 mM potassium phosphate buffer (pH 7.5). (TIF) [file pone.0067197.s003.tif]

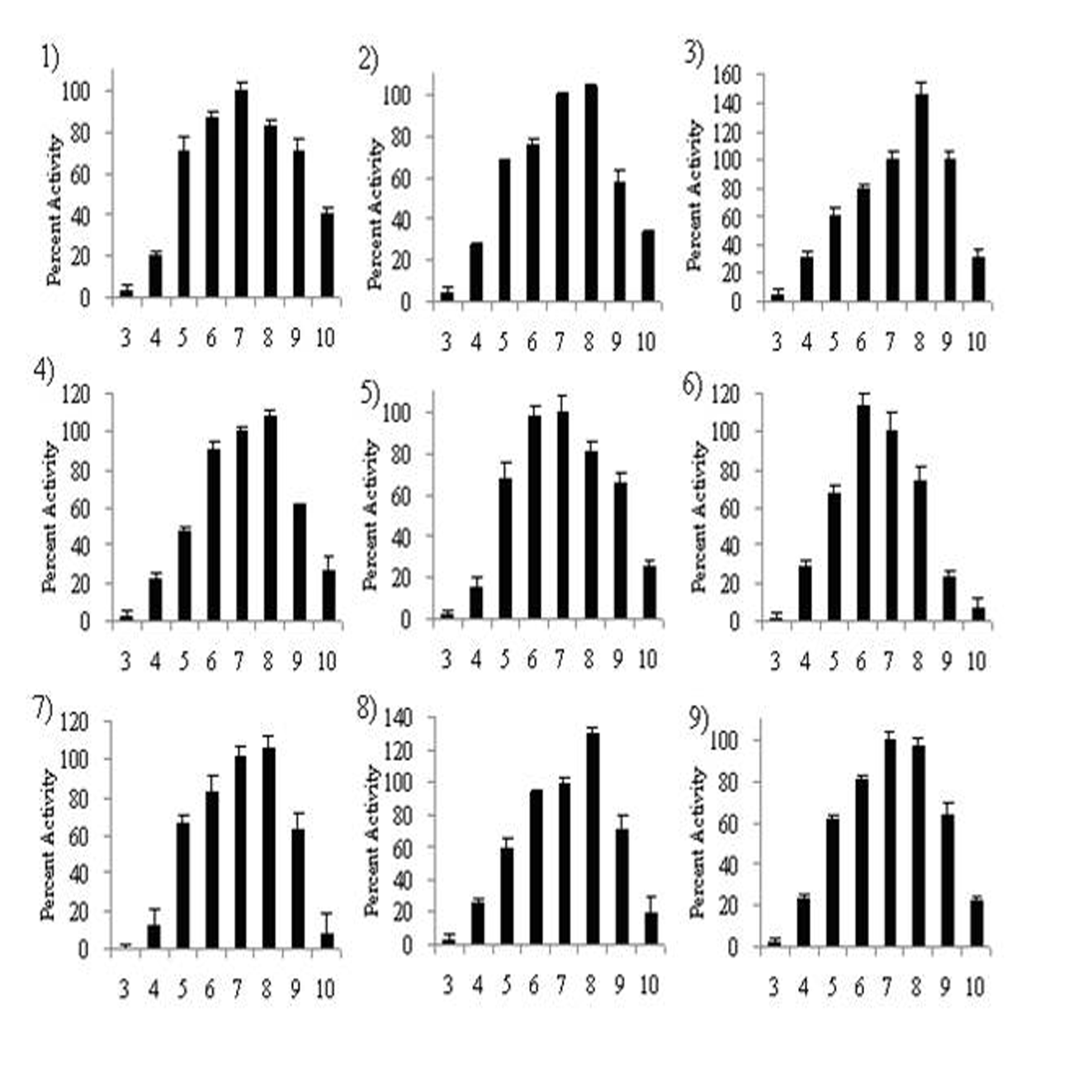

Supplement: Figure S4 — pH activity profile of 1) AcN 2) AkN 3) ApN 4) BgN 5) GpN 6) KpN 7) RjN 8) RkN 9) TpN. Data is normalized to activity at pH 7.0 for each enzyme. Error bars represent the standard deviation from three separate trials. (TIF) [file pone.0067197.s004.tif]

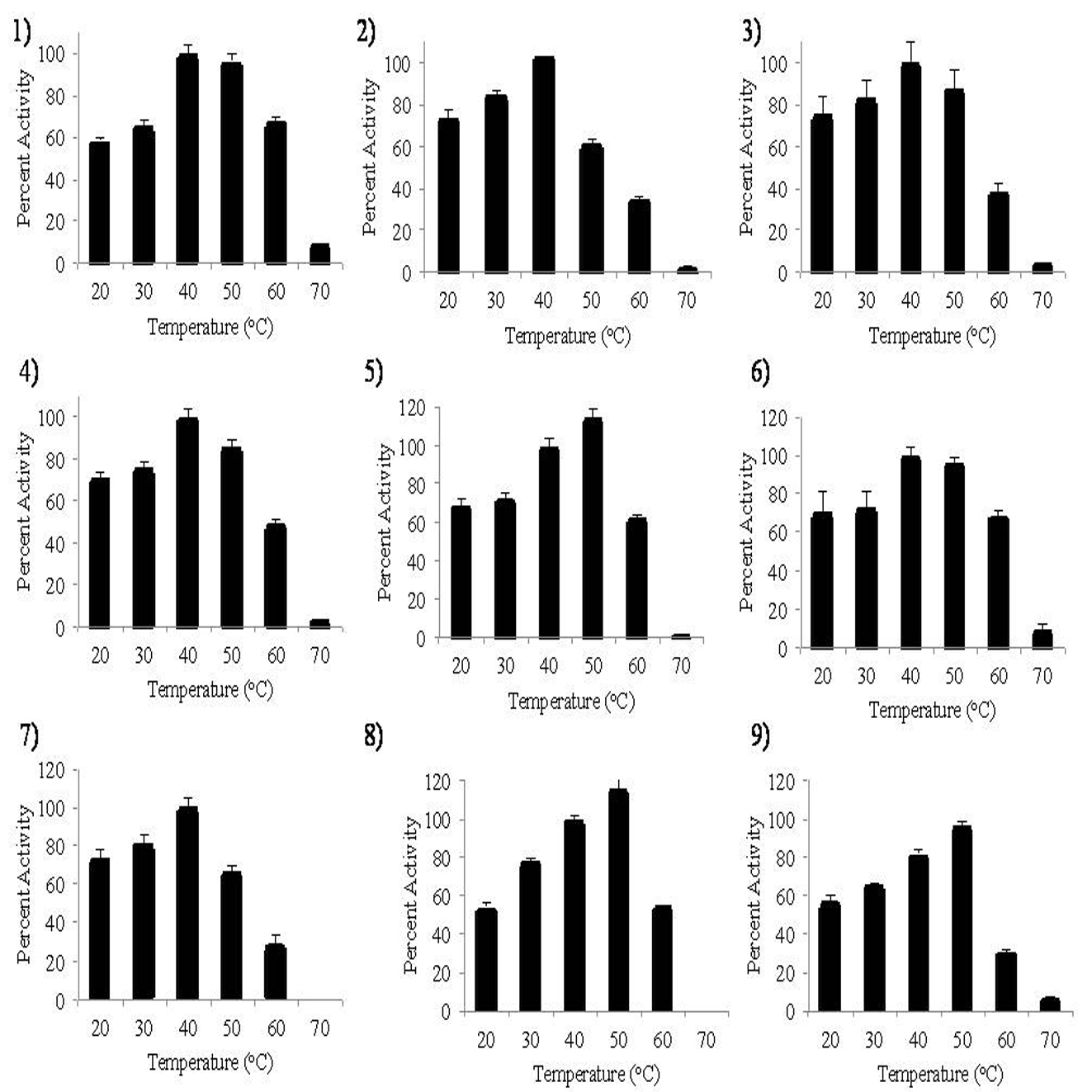

Supplement: Figure S5 — Temperature profile of 1) AcN 2) AkN 3) ApN 4) BgN 5) GpN 6) KpN 7) RjN 8) RkN 9) TpN for IV. Data is normalized to activity at 40°C. Error bars represent the standard deviation from three separate trials. (TIF) [file pone.0067197.s005.tif]

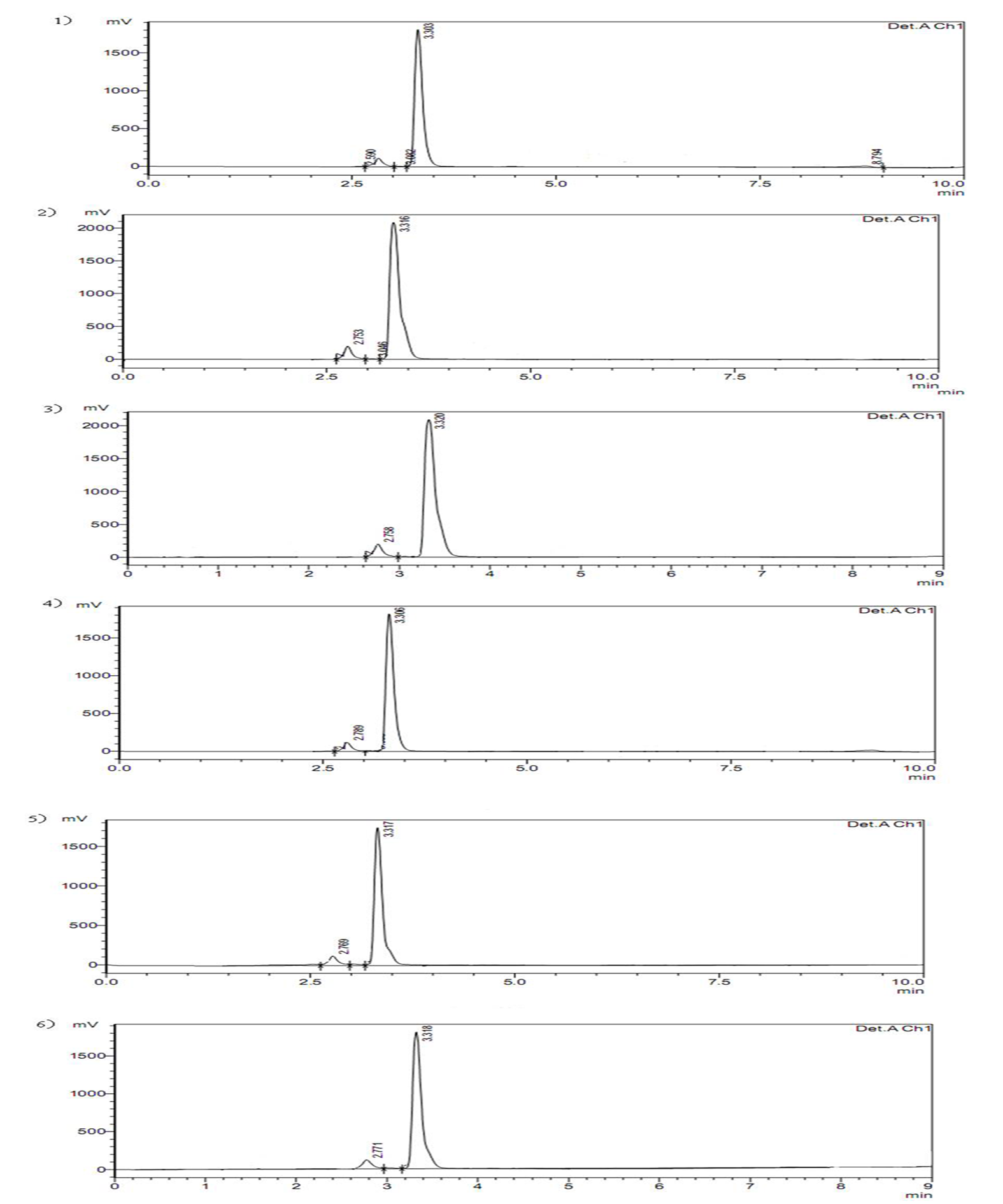

Supplement: Figure S6 — HPLC spectrums of nitrilases which demonstrated no activity for IDAN hydrolysis assay. 1) ApN 2) BgN 3) GpN 4) KpN 5) RjN and 6) TpN. The retention times for IDAN, CCA and IDA peaks were 3.4, 4.2, and 8.1 minutes, respectively. (TIF) [file pone.0067197.s006.tif]

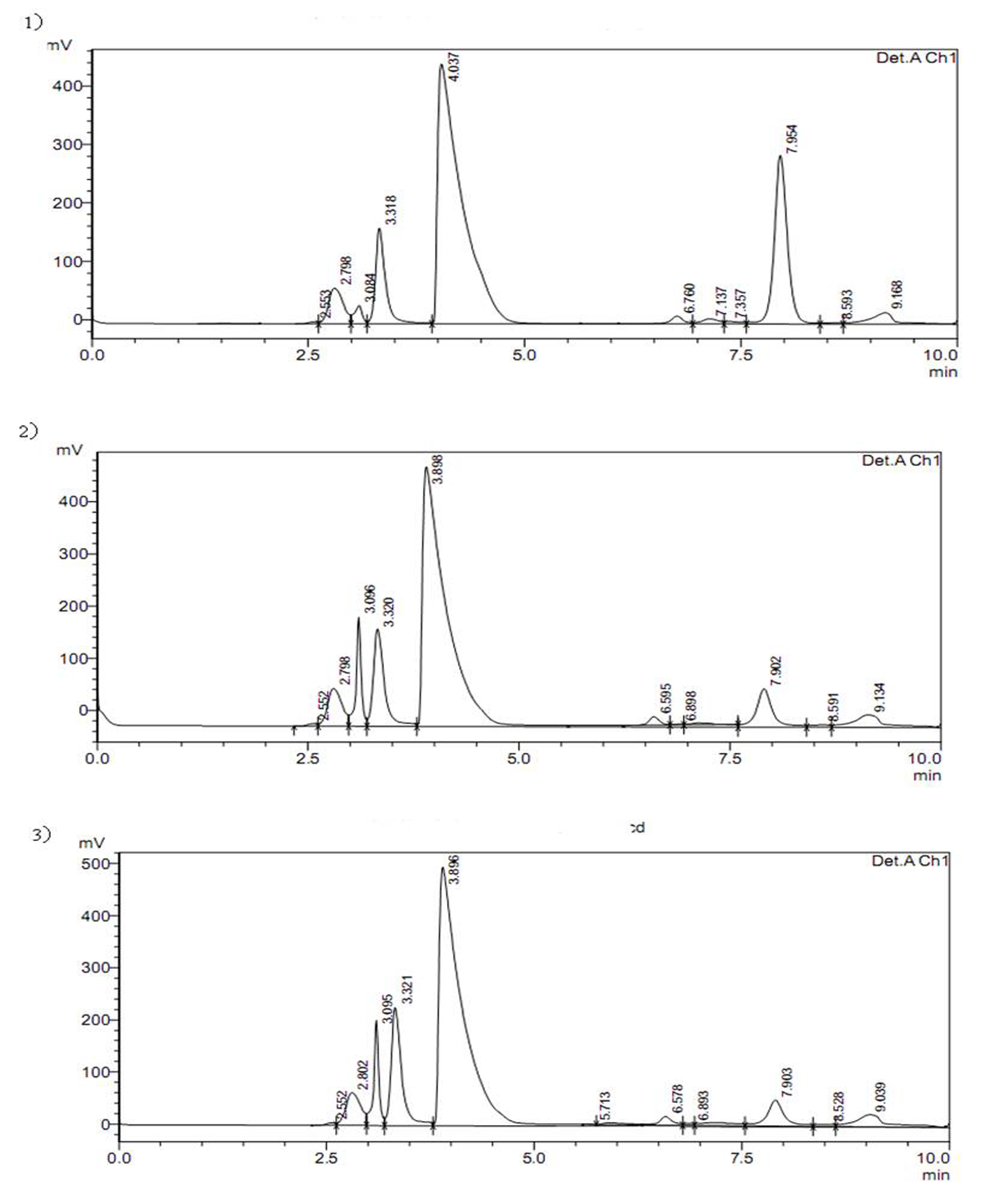

Supplement: Figure S7 — HPLC spectrums of nitrilases which demonstrate IDAN hydrolysis activity. 1) AcN, 2) AkN and 3) RkN. The retention times for IDAN, CCA and IDA peaks were 3.4, 4.2, and 8.1 minutes, respectively. (TIF) [file pone.0067197.s007.tif]

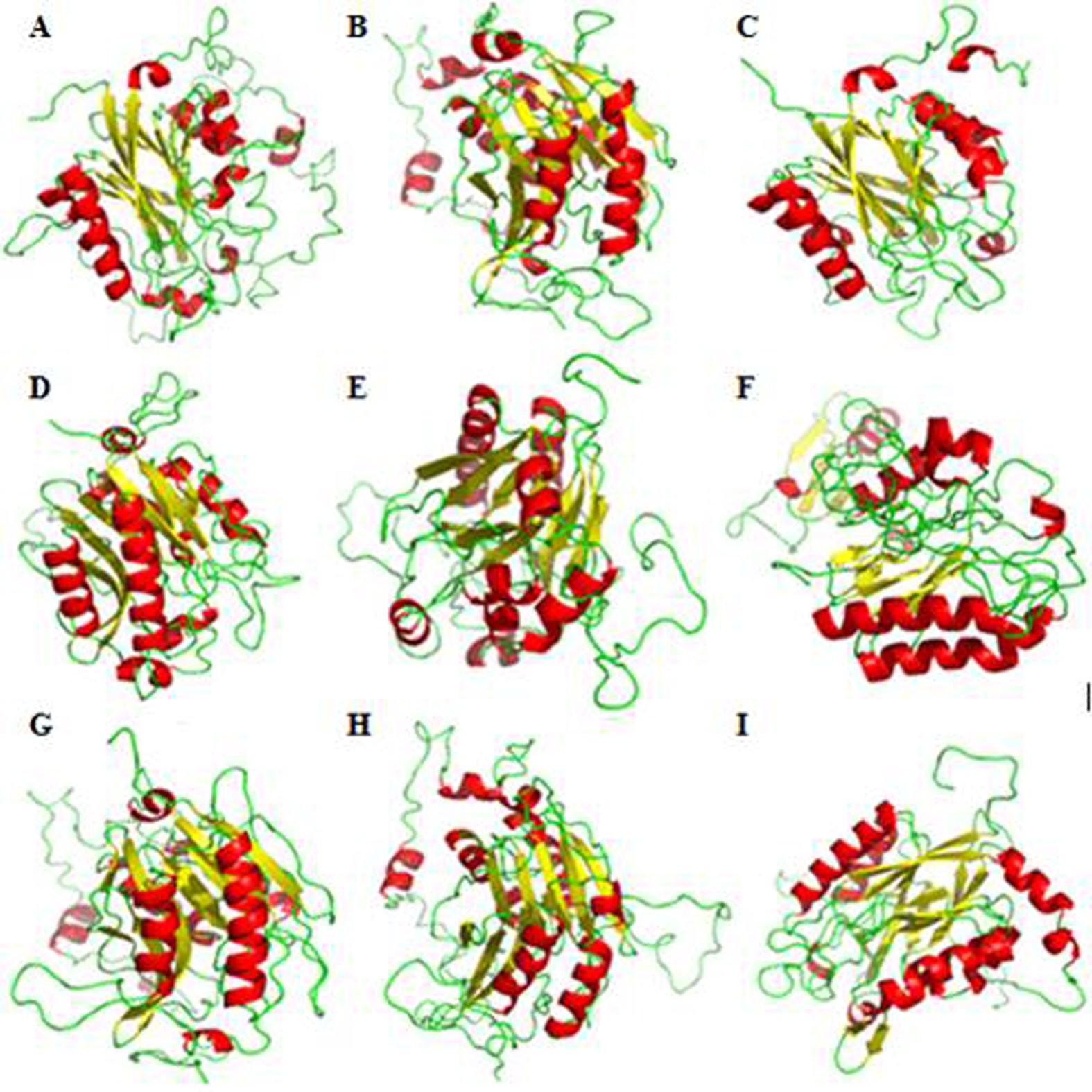

Supplement: Figure S8 — Homology protein models of nitrilases. A) AcN B) ApN C) BgN D) GpN E) RjN F) AkN G) RkN H) KpN and I) TpN. Helix, sheet, loop are displayed in red, yellow and green, respectively. (TIF) [file pone.0067197.s008.tif]

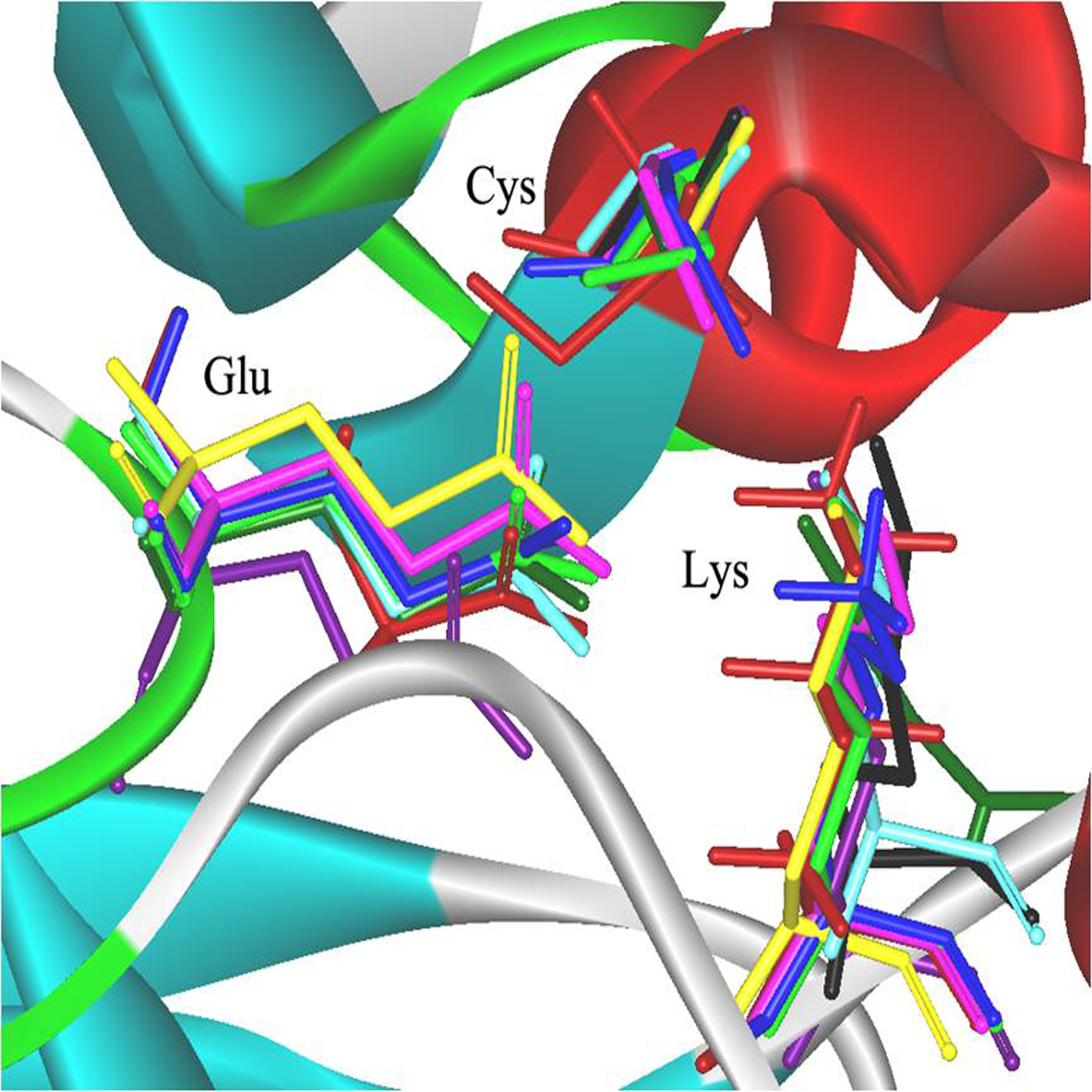

Supplement: Figure S9 — Alignment of nitrilase catalytic triads. AcN (red), AkN (green), ApN (blue), BgN (green), GpN (pink), KpN (purple), RjN (light blue), RkN (black) and TpN (orange). (TIF) [file pone.0067197.s009.tif]

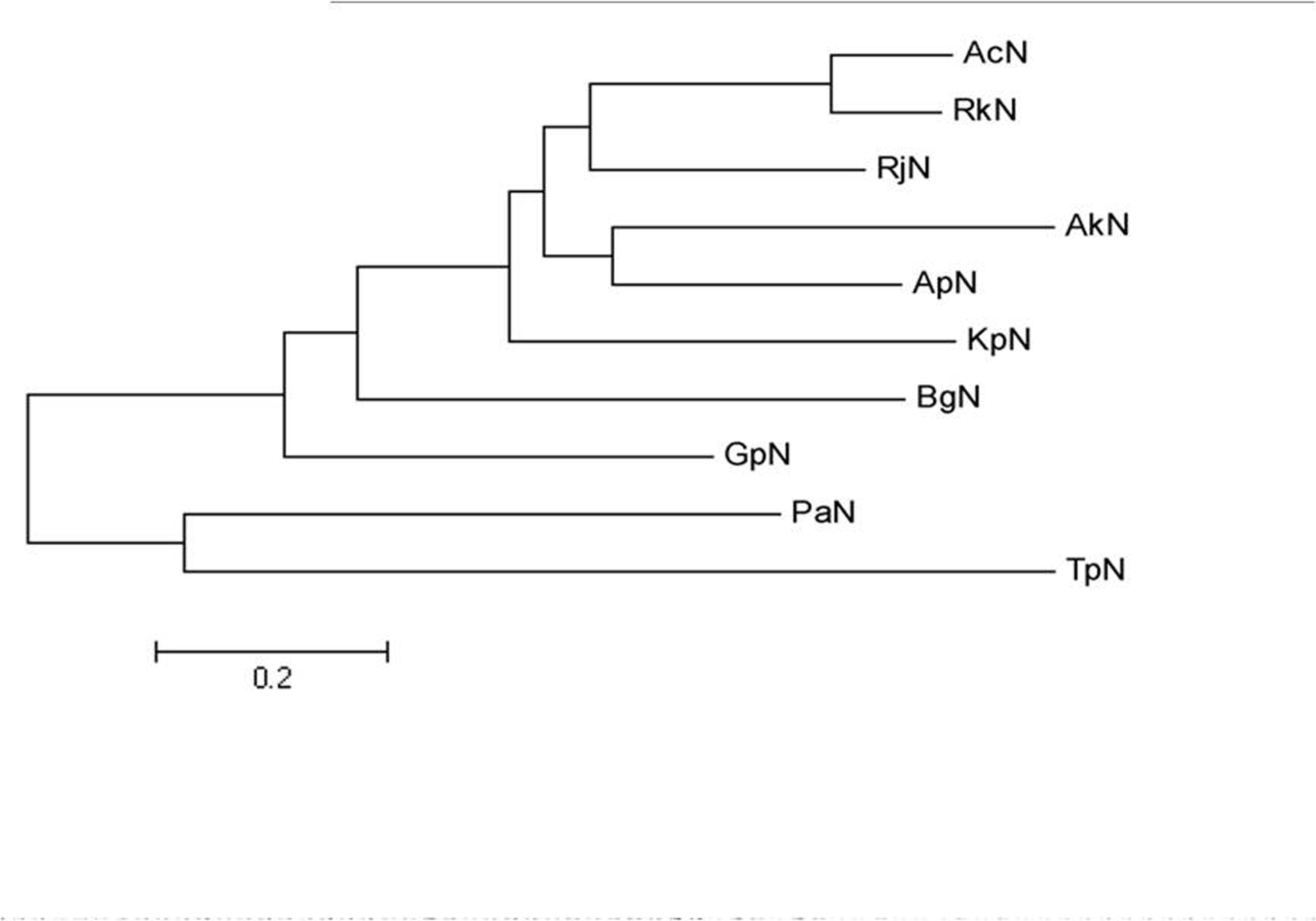

Supplement: Figure S10 — Phylogenetic tree for the nitirlases used in this study based on the sequences identity. (TIF) [file pone.0067197.s010.tif]
