# Supplementary material for: Screening and Improving the Recombinant Nitrilases and Application in Biotransformation of Iminodiacetonitrile to Iminodiacetic Acid
Source: PLoS One. 2013 Jun 27;8(6):e67197. doi: 10.1371/journal.pone.0067197 (PMC3695085; doi:10.1371/journal.pone.0067197)
Supplement: Table S1 — Primers used for PCR amplification of nitrilase genes. (DOC) [file pone.0067197.s011.doc]

Table S1. Primers used for PCR amplification of nitrilase genes.

| Primer | Sequence |
| --- | --- |
| AcN(F) | 5’- AATGGATCCATGGTTTCGTATAACAGCAAG -3’ |
| AcN(R) | 5’- AGGGTCGACCTACTTTGCTGGGACCGG -3’ |
| AkN(F) | 5’- AATGGATCCATGCAGACAAGAAAAATCGTC -3’ |
| AkN(R) | 5’- AGGGTCGACTCAGGACGGTTCTTGCAC -3’ |
| BgN(F) | 5’- AATGGATCCATGAAAGTTGTCAAAGCCG -3’ |
| BgN(R) | 5’- AGGGTCGACTCAGCGCGAACCTGC -3’ |
| KpN(F) | 5’- AATGGATCCATGACCACTTTCAAAGCAGC -3’ |
| KpN(R) | 5’- AGGGTCGACTTAGGAATGTCCGCAATAAC -3’ |
| GpN(F) | 5’- AATGGATCCATGGAGGGGAAGAATATGTC -3’ |
| GpN(R) | 5’- AGGGTCGACTTAATTTTTCCACTCAATAGTTG -3’ |
| RkN(F) | 5’- AATGGATCCATGTCCAGCAATCCAGAGCTC -3’ |
| RkN(R) | 5’- AGGGTCGACCTAGGCCTCCGCCTTGG -3’ |
| RjN(F) | 5’- AATGGATCCATGGTCGAATACACAAACAC -3’ |
| RjN(R) | 5’- AGGGTCGACTCAGATGGAGGCTGTCG -3’ |
| TpN(F) | 5’- AATGGATCCATGGCAGCTTCTTCCACATC -3’ |
| TpN(R) | 5’- AGGGTCGACCTAATTGACATCACCACTTCCC -3’ |
| ApN(F) | 5’- AATGGATCCATGGACCACCCCAAGTTC -3’ |
| ApN(R) | 5’- AGGGTCGACTCAGATCCCTTCCTGATCTG -3’ |

Note: sequences underlined indicated the endonuclease sites of *Nco*I and *Xho*I.
