# Supplementary material for: Screening and Improving the Recombinant Nitrilases and Application in Biotransformation of Iminodiacetonitrile to Iminodiacetic Acid
Source: PLoS One. 2013 Jun 27;8(6):e67197. doi: 10.1371/journal.pone.0067197 (PMC3695085; doi:10.1371/journal.pone.0067197)
Supplement: Table S2 — Primers used for site directed mutagenesis of AcN mutants. (DOC) [file pone.0067197.s012.doc]

Table S2. Primers used for site directed mutagenesis of AcN mutants.

| Mutant | Primer |
| --- | --- |
| F168V(F) | 5’- GAGCACGTTCAGCCGCTGTCCAAAT -3’ |
| F168V(R) | 5’- CGGCTGAACGTGCTCCCAGCAGTTC -3’ |
| L201N(F) | 5’- TTTCAAAACTCCATCGAGGCTAATGC -3’ |
| L201N(R) | 5’- ATGGAGTTTTGAAACACGTCCGGTTG -3’ |
| N127R(F) | 5’- GTTGCTAGACGTCGTAAACTGAAACCA -3’ |
| N127R(R) | 5’- ACGACGTCTAGCAACGATTTCACCACG -3’ |
| S192F(F) | 5’- CCGGCTATGTTCCCGCTGCAACCGGA -3’ |
| S192F(R) | 5’GCGGGAACATAGCCGGCCAAGAAGCG3’ |
| S192H(F) | 5’CCGGCTATGCACCCGCTGCAACC3’ |
| S192H(R) | 5’GCGGGTGCATAGCCGGCCAAGAA3’ |
| C164F(F) | 5’GGTCTGAACTTCTGGGAGCACGTTC3’ |
| C164F(R) | 5’TGCTCCCAGAAGTTCAGACCACCAAC3’ |

Note: sequence underlined indicated the substituted nucleotide.
