# Supplementary material for: Screening and Improving the Recombinant Nitrilases and Application in Biotransformation of Iminodiacetonitrile to Iminodiacetic Acid
Source: PLoS One. 2013 Jun 27;8(6):e67197. doi: 10.1371/journal.pone.0067197 (PMC3695085; doi:10.1371/journal.pone.0067197)
Supplement: Table S3 — Comparison similarity of nitrilases with different protein sequences. (DOC) [file pone.0067197.s013.doc]

Table S3. Comparison similarity of nitrilases with different protein sequences.

|  | PaN | AcN | AkN | BgN | KpN | GpN | RkN | RjN | ApN | TpN |
| --- | --- | --- | --- | --- | --- | --- | --- | --- | --- | --- |
| PaN | 100.0 | 18.7 | 14.5 | 13.7 | 17.9 | 17.1 | 20.6 | 17.9 | 14.1 | 15.6 |
| AcN |  | 100.0 | 37.4 | 29.3 | 38.4 | 29.4 | 68.3 | 51.1 | 48.7 | 13.9 |
| AkN |  |  | 100.0 | 29.3 | 31.8 | 28.2 | 36.0 | 38.2 | 49.5 | 14.9 |
| BgN |  |  |  | 100.0 | 31.7 | 34.3 | 30.3 | 33.3 | 31.1 | 11.3 |
| KpN |  |  |  |  | 100.0 | 27.6 | 34.4 | 39.5 | 39.2 | 11.1 |
| GpN |  |  |  |  |  | 100.0 | 30.4 | 31.6 | 31.9 | 15.8 |
| RkN |  |  |  |  |  |  | 100.0 | 48.7 | 50.2 | 13.3 |
| RjN |  |  |  |  |  |  |  | 100.0 | 49.5 | 12.4 |
| ApN |  |  |  |  |  |  |  |  | 100.0 | 12.8 |
| TpN |  |  |  |  |  |  |  |  |  | 100.0 |
