# Supplementary material for: Screening and Improving the Recombinant Nitrilases and Application in Biotransformation of Iminodiacetonitrile to Iminodiacetic Acid
Source: PLoS One. 2013 Jun 27;8(6):e67197. doi: 10.1371/journal.pone.0067197 (PMC3695085; doi:10.1371/journal.pone.0067197)
Supplement: Table S4 — The expected and experimental molecular weights of nine nitrilases. (DOC) [file pone.0067197.s014.doc]

Table S4. The expected and experimental molecular weights of nine nitrilases.

|  | Expected MW (kDa) | Experimental MW (kDa) |
| --- | --- | --- |
| AcN | 42.4 | 43 |
| AkN | 39.8 | 42 |
| ApN | 30.7 | 32 |
| BgN | 33.3 | 35 |
| GpN | 36.0 | 36 |
| KpN | 39.0 | 40 |
| RjN | 42.6 | 43 |
| RkN | 44.7 | 44 |
| TpN | 35.2 | 34 |
