# Supplementary material for: Screening and Improving the Recombinant Nitrilases and Application in Biotransformation of Iminodiacetonitrile to Iminodiacetic Acid
Source: PLoS One. 2013 Jun 27;8(6):e67197. doi: 10.1371/journal.pone.0067197 (PMC3695085; doi:10.1371/journal.pone.0067197)
Supplement: Table S5 — Melting temperatures of nitrilases used in this study as determined by CD. (DOC) [file pone.0067197.s015.doc]

Table S5. Melting temperatures of nitrilases used in this study as determined by CD

| Nitrilase | T_m_ (°C) |
| --- | --- |
| AcN | 57.2 |
| AkN | 46.8 |
| ApN | 55.6 |
| BgN | 51.0 |
| GpN | 51.8 |
| KpN | 56.0 |
| RjN | 48.0 |
| RkN | 55.1 |
| TpN | 52.2 |
