# Supplementary material for: Screening and Improving the Recombinant Nitrilases and Application in Biotransformation of Iminodiacetonitrile to Iminodiacetic Acid
Source: PLoS One. 2013 Jun 27;8(6):e67197. doi: 10.1371/journal.pone.0067197 (PMC3695085; doi:10.1371/journal.pone.0067197)
Supplement: Table S6 — Docking analysis of AcN, AfN and RkN with IDAN. (DOC) [file pone.0067197.s016.doc]

Table S6. Docking analysis of AcN, AfN and RkN with IDAN.

|  |  | AcN | AfN | RkN |
| --- | --- | --- | --- | --- |
| Distances(Å)  between IDAN and NIT | N to –SH of Cys | 2.2 (H-bond) | 2.8 (H-bond) | 2.3 (H-bond) |
|  | N to -NH of Lys | 1.8 (H-bond) | 2.5 (H-bond) | 2.9 (H-bond) |
|  | N to -OH of Glu | 2.9 (H-bond) | 3.4 | 3.5 |
| Distances(Å)  between CCA and NIT | N to –SH of Cys | 2.5 (H-bond) | 2.7 (H-bond) | 2.6 (H-bond) |
|  | N to -NH of Lys | 2.4 (H-bond) | 3.3 | 3.3 |
|  | N to -OH of Glu | 3.0 (H-bond) | 3.3 | 3.5 |
| Docked energy to IDAN(E_i_) | (kcal/mol) | -3.157 | -2.473 | -2.134 |
| Docked energy to CCA(E_C_) | (kcal/mol) | -2.656 | -1.640 | -1.058 |
| Ratio(E_C_/E_i_) | - | 84.1% | 66.3% | 49.6% |
